# Maternal Magnesium Sulfate Supplementation in a Pre-Farrow Diet Improves Factors Important for Piglet Viability

**DOI:** 10.3390/ani8100185

**Published:** 2018-10-22

**Authors:** Kate Plush, Alice Weaver, Lauren Staveley, William van Wettere

**Affiliations:** School of Animal and Veterinary Science, The University of Adelaide, Roseworthy 5371, SA, Australia; alice_weaver@outlook.com (A.W.); lauren.staveley@adelaide.edu.au (L.S.); william.vanwettere@adelaide.edu.au (W.v.W.)

**Keywords:** hypoxia, magnesium, colostrum, thermoregulation, vitality, piglet survival

## Abstract

**Simple Summary:**

The number of pigs sold per sow per year is a key determinant of productivity and therefore, profitability of pork production. This is currently limited by high levels of piglet mortality prior to weaning, with lack of oxygen during the birth process a primary cause of piglet death and failure to thrive. This experiment investigated the effect of adding magnesium sulphate to sow diets fed during the last five days of pregnancy on piglet survival and measures of viability at, and soon after, birth. The addition of magnesium sulphate to sow diets resulted in piglets which showed signs of increased viability and vitality immediately after birth, with the beneficial effects most evident in ‘at risk’ piglets. These data suggest that magnesium sulphate supplementation at the end of gestation represents a commercially viable solution to reduce the negative effects of the birth process on piglet viability, which may in turn improve survival.

**Abstract:**

Piglet hypoxia during farrowing is common and can lead to increased stillbirth rates, reduced piglet vitality, and an increased risk of preweaning mortality. Magnesium sulfate (MgSO_4_) has successfully been used as a neuroprotectant and readily crosses the placenta in humans. Based on this human data, the aim of this study was to determine if maternal dietary supplementation with MgSO_4_ prior to farrowing would reduce the impact of piglet hypoxia during the peri-natal period. Five days prior to farrowing, Large White × Landrace sows were fed either standard lactation sow diet (Cont; n = 30) or lactation diet supplemented to deliver 21 g/day MgSO_4_ (Mg; n = 31). There was no effect of treatment on the percentage of stillborn piglets (*p* > 0.05). However, Mg piglets tended to have higher vitality scores immediately after birth (*p* < 0.10), were quicker to suck, and had higher day one blood glucose concentrations when compared with Cont piglets (*p* < 0.05). Furthermore, hypoxic piglets born to Cont sows did not gain weight from birth to 24 h, but Mg piglets did (*p* < 0.05), suggesting improved colostrum ingestion. In conclusion, MgSO_4_ may reduce the negative impacts of birth hypoxia, improving piglet vitality, and colostrum intake during the peri-natal period.

## 1. Introduction

Following the introduction of the farrowing crate, there has been little improvement in reducing piglet mortality prior to weaning. Conservative reports of pre-weaning mortality state a 5.4% incidence of peri-partum death (stillbirth) resulting from anoxia, and that more than 11% of piglets die after parturition [1]. Non-lethal hypoxic damage caused during farrowing contributes to this post-partum mortality indirectly by reducing the vitality of the piglet, and impairing thermoregulation [2]. Even if these impairments do not result in death directly, they will increase the length of time the piglet spends in proximity to the sow for milk access and warmth, and so the risk of sow overlay. Current methods of reducing peri-natal piglet mortality caused by anoxia and hypoxia include increased supervision at farrowing and the culling of older sows that are more likely to display higher stillbirth rates. Both options result in a decrease in farrowing house profitability due to increased labor costs, and the replacement of older parity sows with less productive gilts, respectively.

Strategies that prevent, limit, or slow neuronal damage, which occurs during hypoxia, are termed neuroprotectants, and are widely studied in human medicine for the prevention and treatment of central nervous system disease. Magnesium ions are essential for key cellular processes, and can influence cell apoptosis through the reduced production of pro-inflammatory cytokines and free radicals after hypoxia-ischemia, when blood flow is restored after an hypoxic event [3]. Piglets have been used as a model for hypoxia-ischemia and results in this species have demonstrated that pre-treatment with magnesium sulfate (MgSO_4_) results in positive outcomes for neuronal membrane function [4,5]. Eventual neurological outcomes for progeny are also impacted upon as a reduced incidence of gross motor dysfunction [6] and cerebral palsy [7] after maternal infusion of MgSO_4_ in human pre-term babies.

Whilst the investigations above administered MgSO_4_ via infusion, there is evidence in humans to suggest that an oral dose is just as effective [8]. In finisher pigs, dietary supplementation with MgSO_4_ for five days prior to slaughter was successful in increasing plasma Mg concentrations by approximately 10% suggesting the same is true in this species [9]. Magnesium readily crosses the human placenta [10] and fetal blood concentrations correlate well with maternal levels in humans [8]. The pig placenta differs to that of humans in structure (a thicker epitheliochorial versus hemochorial barrier) and whilst little is known about ion placental transfer in livestock species, it is generally accepted that fetal levels of Mg are maintained at higher concentrations than maternal levels [11]. A simple dietary manipulation to increase MgSO_4_ concentrations within the sow around the time of parturition may therefore be an attractive option for reducing the impacts of piglet anoxia and hypoxia on pre-weaning mortality. The aim of this study was to determine if dietary MgSO_4_ supplementation in sow diets prior to farrowing would reduce the impact of hypoxic damage on newborn piglets, improving vitality and growth performance over the peri-natal period.

## 2. Materials and Methods

All animal procedures were conducted with approval from The University of Adelaide’s Animal Ethics Committee under the guide of the Code of Practice for the Care and Use of Animals for Scientific Purposes (approval number S-2013-184).

This study used 61 Large White × Landrace sows and their litters across four farrowing blocks during summer months. Five days prior to the expected farrowing date, sows were moved from group gestational housing to one of two rooms within a farrowing shed. Each room was identical in design, in that it housed 12 farrowing crates, was climate controlled, and shared common ventilation via a walkway that stretched the length of the shed. The farrowing crate that housed each sow measured 2.4 m by 1.8 m and contained a sow feeder and drinker, a heated creep mat, and an infrared heat lamp positioned at the rear of the sow during the farrowing period only.

A standard commercial lactation sow diet (14.2 MJ DE/kg; Lienerts, SA Australia) in the form of mash feed was used as a base to formulate the two dietary treatments. For every 100 kg of feed, the control (Cont; n = 30) diet contained 95 kg of standard lactation sow diet and 5 kg of filler made up of bentonite and mill mix in equal proportions. Treatments were randomly distributed across rooms, such that each room contained sows in both treatment groups.

The diet supplemented with MgSO_4_ (Mg; n = 31) contained 95 kg of standard lactation sow diet, 4.3 kg of filler and 0.7 kg of MgSO_4_ (Epsom salts; Redox, NSW Australia). Sows were fed 1.5 kg twice daily upon entry to the farrowing shed at 0700 and 1600 to give a total allocation of 3.0 kg feed/sow/day. At this inclusion rate and feeding allowance, sows within the Mg treatment group received 21 g MgSO_4_/day (0.7%). The two diets were fed up until the day of farrowing (for 4.4 ± 0.2 days) after which, point sows were fed the standard lactation sow diet ad libitum until weaning.

Each sow was monitored during farrowing and the average inter-piglet birth interval, total farrowing duration, number of stillborn piglets born alive, and total piglets born were recorded.

Piglet fostering was conducted within 24 h of birth and involved the sow being allocated the number of piglets she could feed based on the quantity of functional teats present on her udder. Minimal piglet movement was adopted, and when piglets were moved it was conducted within treatment. All piglets received an intramuscular injectable dose of iron (200 mg) and were tail docked at one day of age.

The following measures were collected from piglets (n = 752); birth order, inter-piglet birth interval as well as a cumulative time since the birth of the first piglet was recorded. A meconium score to indicate birth trauma was allocated with no staining receiving a score 0, some staining receiving a 1, moderate staining receiving a 2, and severe staining receiving a 3 [12]. At this time piglets also received a categorical vitality score, as shown in Table 1, based on the level of movement and breathing after birth [13]. After the two scores were allocated, whole umbilical cord blood was collected and analyzed immediately for lactate, pH, and glucose concentration (Epoc, Alere QLD Australia). If the cord was broken at the time of expulsion this was recorded, and if intact, the cord was broken close to the sow in order to collect the sample. The latency for the piglet to contact any part of the sow’s udder, and successfully suck on a teat to receive colostrum was timed (and recorded in minutes). If the piglet failed to reach one of these behavioral milestones they received the maximum time of 3 h (360 min). Fifteen minutes following birth, the piglet was removed from the farrowing crate, sexed, weighed, and recorded for rectal temperature. This procedure took no more than 1 min per piglet, and after it was conducted, the piglet was placed in the identical position in the farrowing crate from which it was removed.

At one day of age, all piglets were weighed and recorded for rectal temperature once more. A 3 mL blood sample was collected from the jugular vein using a 23 G needle and syringe and then transferred into a serum collection tube. Blood glucose was measured immediately (Hemocue, NSW Australia) and samples were then left overnight at 4 °C. The following day, the samples were centrifuged and serum was removed from the blood tube and used to estimate colostrum ingestion and absorption using the immunocrit test [14]. This procedure used ammonium sulfate ((NH_4_)_2_SO_4_) to precipitate proteins present in serum, largely consisting of maternally derived immunoglobulins (Ig) from colostrum. To summarize, 100 µL of serum was mixed with 100 µL of 40% (NH_4_)_2_SO_4_ solution, drawn into a hematocrit micro-capillary tube, and centrifuged at 3000 *g* for 5 min. The length of the precipitate present at the bottom of the tube was then divided by the total volume of solution to give a proportion reading.

Data were analyzed in GenStat 16th edition (VSN International, Hemel Hempstead, UK). For sow measures, an unbalanced design ANOVA was used, and the model included the fixed effects of replicate (1 to 4), farrowing room (1 or 2), sow parity (2 to 9), treatment (Cont, Mg), and the interaction between parity and treatment. Piglet measures were analyzed using a linear mixed model with sow as the random effect, litter size as a covariate, and the fixed effects of replicate (1 to 4), farrowing room (1 or 2), sow parity (2 to 9), sex (male, female), weight grade (light < 0.8 kg, average 0.8 to 1.8 kg, heavy > 1.8 kg), birth order grade (first 1 to 4, middle 5 to 8, and last > 8), meconium stain score (0 to 3), treatment (Cont, Mg), and any significant two way interactions between these main effects. Piglet behaviors were log_10_ transformed to normalize distribution.

## 3. Results

There was no difference in total number of piglets born, number of piglets born alive, number of stillborn piglets, or inter-piglet birth interval between the two treatments, as shown in Table 2 (*p* > 0.05). Total farrowing duration was increased by 1.2 h in Mg sows when compared with Cont, as shown in Table 2 (*p* < 0.05).

Inter-piglet birth interval was affected by litter size (*p* < 0.001), with an increase in one piglet born alive resulting in a decrease in the interval of 2.9 ± 0.7 min. The only other factor shown to increase birth interval was piglet weight, with heavier piglets resulting in a longer interval, as shown in Table 3 (*p* < 0.01). Cumulative birth duration was increased in heavy piglets, those born last in the birth order, and those with a higher stain score, as shown in Table 3 (*p* < 0.05). A positive relationship between cord blood lactate concentration and litter size was identified. For an extra piglet born alive, an increase of 0.18 ± 0.10 mmol/L cord lactate was observed (*p* < 0.001). Cord lactate levels were highest in average weight, last born, and high stain score piglets, as shown in Table 3 (*p* < 0.05). Cord pH was lowest in light weight and high stain score piglets but remained unaffected by birth order, as shown in Table 3 (*p* < 0.05). Glucose concentrations in the cord blood tended to be lower in light piglets (*p* < 0.1), were lowest in those born later in the birth order (*p* < 0.05), but highest in piglets with a stain score of 3, as shown in Table 3 (*p* < 0.05).

**Figure 1:**
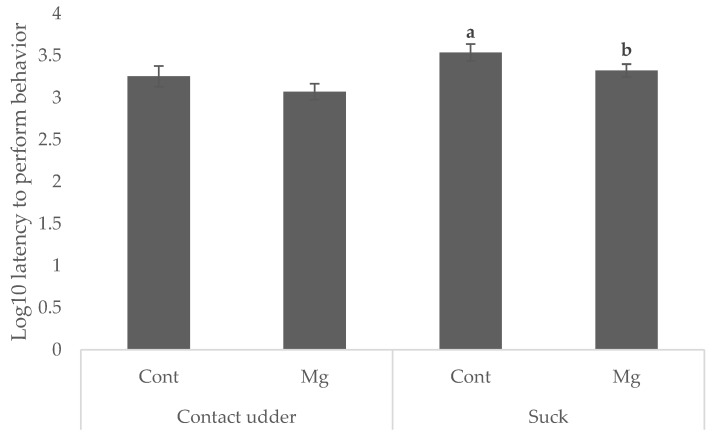
Mean ± s.e.m log_10_ latency (in minutes) to contact the sow’s udder and successfully suck for piglets born to sows fed a standard lactation diet (Cont) and those supplemented with 21 g/day MgSO_4_ (Mg) prior to farrowing. ^a,b^ Means within behavioral milestone not having the same superscript are significantly different (*p* < 0.05).

**Figure 2:**
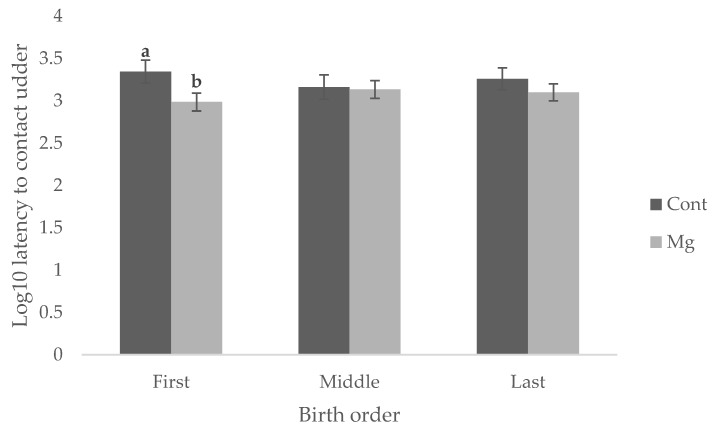
Mean ± s.e.m log_10_ latency (in minutes) to contact the sow’s udder for piglets first (1–4), middle (5–8), or last (>8) in the birth order from sows fed a standard lactation diet (Cont) and those supplemented with 21 g/day MgSO_4_ (Mg) prior to farrowing. ^a,b^ Means within birth order not having the same superscript are significantly different (*p* < 0.05).

Piglets born to Mg sows were quicker to suck (35.1 min) than those born to Cont (42.1 min and 57.5 min, respectively) as shown in Figure 1 (*p* < 0.05). Whilst there was no significant main effect of treatment on latency to contact the udder, as shown in Figure 1 (*p* = 0.119), Mg piglets born first in the birth order reached this behavioral milestone faster (16.1 min) than Cont piglets born first (36.8 min) as shown in Figure 2 (*p* < 0.05). No difference was observed when piglets were born middle or last in the birth order, as shown in Figure 2 (*p* > 0.05).

**Figure 3:**
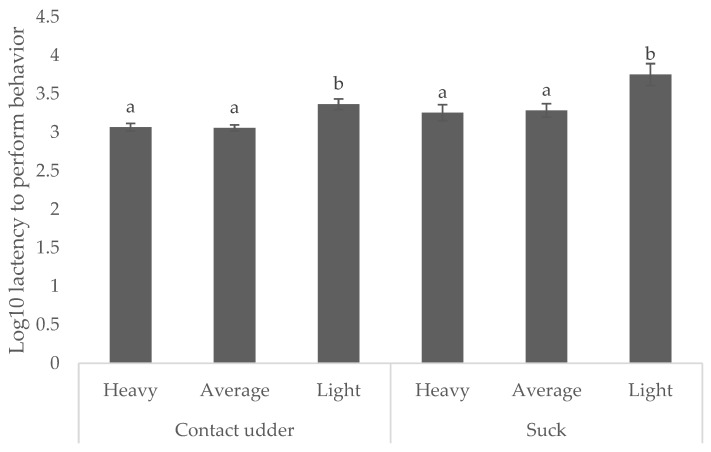
Mean ± s.e.m log_10_ latency to contact the sow’s udder and successfully suck for piglets classed as heavy (>1.8 kg), average (0.8 to 1.8 kg), and light (<0.8 kg). ^a,b^ Means within birth order not having the same superscript are significantly different (*p* < 0.05).

Light weight piglets were consistently slower to achieve both behavioral milestones. Those weighing < 0.8 kg took 38.1 min to contact the udder (compared with 19.1 and 19.5 min for average and heavy, respectively) and 93.9 min to successfully suck (compared with 32.1 min and 38.6 min for average and heavy piglets, respectively,) as shown in Figure 3 (*p* < 0.05).

There was a tendency for piglets from the Mg treatment to display higher vitality (1.6 ± 0.1: *p* < 0.1) than Cont piglets (1.4 ± 0.1). Vitality score was affected by weight, with light piglets (<0.8 kg; 1.7 ± 0.2) receiving a lower score than average (0.8 to 1.8 kg; 2.2 ± 0.1) and heavy (>1.8 kg; 2.3 ± 0.2) piglets (*p* < 0.001). Additionally, piglets receiving the highest stain score of 3 displayed reduced vitality after birth (1.1 ± 0.2; *p* < 0.001) when compared with other scores (score 0: 1.8 ± 0.2, score 1: 1.6 ± 0.1, score 2: 1.6 ± 0.2). No other effects impacted piglet vitality (*p* > 0.05).

**Figure 4:**
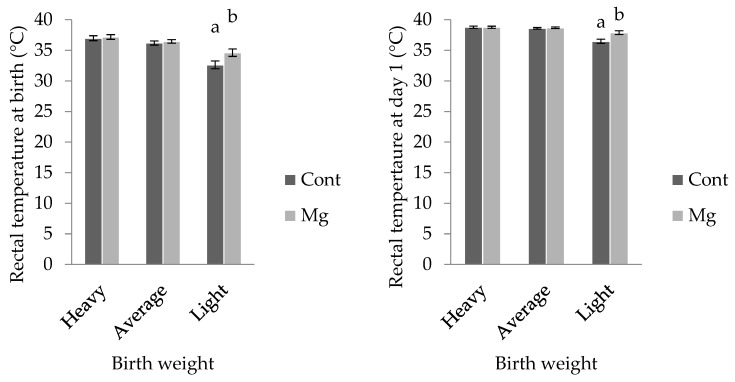
Mean ± s.e.m rectal temperature (°C) at birth and at one day of age for piglets classed as heavy (>1.8 kg), average (0.8 kg to 1.8 kg), and light (<0.8 kg) from sows fed a standard lactation diet (Cont) and those supplemented with 21 g/day MgSO_4_ (Mg) prior to farrowing. ^a,b^ Means within weight on day of measure not having the same superscript are significantly different (*p* < 0.05).

For light weight piglets, rectal temperature was higher in Mg piglets (34.6 ± 0.6 °C) when compared with Cont (32.6 ± 0.6 °C; *p* < 0.05); however, this treatment effect was not observed in the other weight categories, as shown in Figure 4 (*p* > 0.05). Similarly, when taken at one day of age, rectal temperature was higher in light Mg piglets (37.9 ± 0.3 °C) when compared to light Cont piglets (36.5 ± 0.3 °C; *p* < 0.001), as shown in Figure 4. There was no effect of piglet sex on rectal temperature at birth, however at one day of age, females recorded a higher value (38.4 ± 0.1 °C) than males (38.2 ± 0.1 °C; *p* < 0.05). No other factors were shown to impact the rectal temperature of piglets (*p* > 0.05).

There was a tendency for Mg piglets to record higher glucose levels (5.9 ± 0.4 mmol/L) than Cont (5.3 ± 0.3 mmol/L; *p* < 0.1). Light weight piglets (<0.8 kg) were identified as having reduced blood glucose concentrations on day one (3.7 ± 0.9 mmol/L) when compared with average (0.8 to 1.8 kg: 6.1 ±0.2 mmol/L) and heavy (>1.8 kg: 6.6 ± 0.4 mmol/L) piglets (*p* < 0.05). No other significant factors were identified for blood glucose concentration (*p* > 0.05). Light weight piglets (<0.8 kg) recorded a significantly lower immunocrit reading (0.08 ± 0.2) than average (0.8 kg to 1.8 kg: 0.12 ± 0.01) and heavy (>1.8 kg: 0.13 ± 0.01) piglets (*p* < 0.05) when measured at one day of age. No other effects influenced immunocrit (*p* > 0.05).

**Figure 5:**
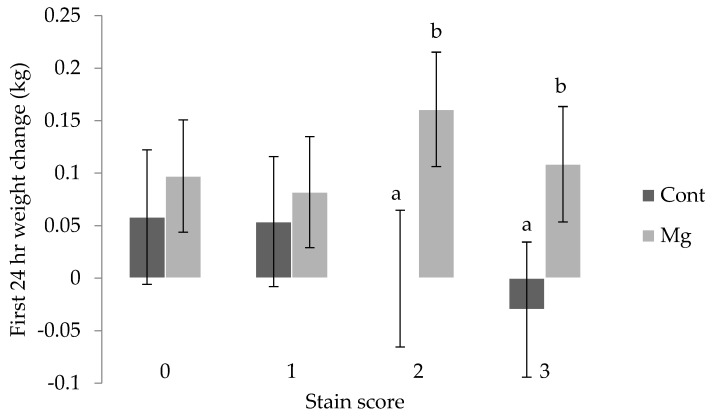
Mean ± s.e.m weight change (kg) over the first 24 h following birth for piglets with no staining receiving a score 0, some staining receiving a 1, moderate staining receiving a 2, and severe staining receiving a 3, from sows fed a standard lactation diet (Cont) and those supplemented with 21 g/day MgSO_4_ (Mg) prior to farrowing. ^a,b^ Means within stain score not having the same superscript are significantly different (*p* < 0.05).

Piglet weight change in the 24 h after birth was influenced by weight at birth (*p* < 0.01), with those defined as heavy (>1.8 kg) and average (0.8 to 1.8 kg) gaining weight (0.11 ± 0.05 kg, 0.13 ± 0.04 kg, respectively), and light piglets (<0.8 kg) effectively recording no change (−0.03 ± 0.06 kg). The interaction between stain score and treatment was also shown to be significant for weight change over the first day, with piglets from the Mg treatment receiving a stain score of 2 or 3 gaining weight (0.16 ± 0.05 kg and 0.11 ± 0.06 kg), and the weight of Cont piglets within these scores remaining constant (0.00 ± 0.06 kg and -0.03 ± 0.06 kg, respectively) as shown in Figure 5 (*p* < 0.01).

## 4. Discussion

This research highlights that behavioral milestones were achieved quicker when piglets were born to Mg supplemented sows, and weight gain in the first day was higher in this treatment when piglets experienced hypoxia. Additionally, Mg supplementation appeared to be of benefit to light weight piglets, with significant improvements in thermoregulation at birth and on day 1 in these animals. There was some evidence that Mg supplementation also improved vitality and glucose concentrations across all piglets. No detrimental effect of Mg supplementation was observed on any piglet measure, and so these results suggest that the inclusion on MgSO_4_ in a pre-farrow diet, at the levels currently tested, is of benefit for piglet viability, especially for hypoxic and light weight piglets, which are at increased risk of mortality.

Whilst there was no impact of MgSO_4_ supplementation on inter-piglet birth intervals, the total farrowing duration was increased in this treatment. The laxative effect of MgSO_4_ is well documented, and its dietary inclusion has been shown to reduce the incidence of constipation in sows [15]. Constipation is commonly associated with an increased farrowing duration [16], and so it might have been reasonable to expect a shorter length of parturition in Mg sows. The observed increase in farrowing length in Mg sows was thus unexpected, but may be explained by the increased levels of circulating magnesium acting as calcium-channel blockers in the myometrial tissue [17]. Whilst shown to be ineffective in halting parturition completely in the human literature [18], MgSO_4_ reduced oxytocin-induced contractions by 30–40% in myometrial strips in vitro [19]. The increased farrowing duration bore little impact on the proportion of piglets born dead in the litter, likely explained by the small two-minute inter-piglet interval increase, and so is of little concern with regards to piglet mortality.

With pigs being a polytocous species, the risk of intra-partum hypoxia is high. Extended labor, or compression or rupture of the umbilical cord, can result in a hypoxic event for the piglet. The piglet is born dead if this event is severe enough. In addition to anoxia, non-lethal hypoxic damage caused during labor contributes to mortality indirectly. The neuronal damage that occurs during neonatal hypoxemia is complex, but to simplify a review by Johnston et al. [20], the excito-oxidative cascade that results from oxygen and glucose deprivation causes a release of glutamate to the brain’s extra-cellular space. It is glutamate that opens calcium permeable channels, allowing the influx of this ion into neurons resulting in oxidative stress, edema, lactic acidosis, and eventually cell apoptosis. The exact mechanism by which magnesium acts as a neuroprotectant is unclear as it is involved in so many cellular functions. However, its primary role in the prevention of hypoxic brain damage appears to be in the ability to block one of these calcium permeable channels (NMDA receptor) [21]. In the present study, meconium stain score was a suitable measure of birth hypoxia as the cumulative duration of birth was longer, cord lactate and glucose concentration higher, and cord pH lower in highly stained piglets. Piglets born to MgSO_4_ treated sows with a high stain score were able to gain weight within one day of age whilst those from Cont sows did not. This finding is suggestive of improved colostrum intake over the first 24 h following birth. Such an outcome is difficult to achieve as the piglet must maintain thermoregulation homeostasis, locate the sow’s udder, compete with littermates, and have a sucking reflex; all traits that may be inhibited after a hypoxic event [2]. Whilst blood samples from sows and piglets were not analyzed for circulating magnesium concentrations, this behavioral improvement may be linked to the neuroprotective effect that magnesium exerts during hypoxia. Further work examining the physiology underpinning these changes should be conducted.

Another important determinant of piglet survival is birthweight. Low birthweight piglets are born with lower energy stores, lose heat more rapidly to their environment, and are less competitive when it comes to acquisition of colostrum [22]. In the present investigation, light weight piglets born to Mg sows displayed rectal temperature two degrees higher at birth, and 1.5 degrees higher at one day of age, confirming improved thermoregulatory outcomes in these individuals. Newborn piglets are born with very little subcutaneous fat and are solely reliant on muscular shivering to maintain homeothermic balance until they achieve enteric feeding. The temperature improvement witnessed immediately following birth would suggest that light piglets born to Mg sows were able to fuel shivering more effectively than their Cont counterparts. Whilst no change in mean birthweight was observed in these light piglets (~660 g for both treatments), perhaps skeletal glycogen stores were increased. MgSO_4_ has been shown to dilate human uterine arteries [23] improving blood flow and so nutrient exchange to the growth restricted fetus in late gestation. The tendency for increased circulating blood glucose concentrations in the Mg piglets at one day of age would give support to this notion, but further work examining piglet glycogen stores in skeletal tissue after maternal Mg supplementation should be conducted. The thermoregulatory improvement observed at one day of age is probably explained by a combination of both the aforementioned improved glycogen stores coupled with enhanced energy acquisition (colostrum intake) and utilization (metabolism of fatty acids to drive shivering). 

## 5. Conclusions

Maternal dietary MgSO_4_ supplementation prior to farrowing improves piglet vigor, energy availability, and thermoregulation, with this being especially true for those that are at increased risk of mortality; light weight and hypoxic individuals. Whilst we did not follow these piglets after the perinatal period, the present findings would advocate the use of increased levels of magnesium in a pre-farrow diet in order to improve piglet pre-weaning mortality. For this to be confirmed, further work with larger sample sizes following piglet performance through to weaning is required.

## Figures and Tables

**Table 1:** Categorical scoring system used to subjectively estimate piglet vitality immediately following birth [13].

Vitality Score	Description
0	No movement, no breathing after 15 s
1	No movement after 15 s, piglet is breathing or attempting to breathe
2	Piglet movement within 15 s, breathing or attempting to breathe
3	Good movement and breathing, piglet attempts to stand within 15 s

**Table 2:** Measurements (mean ± s.e.m collected from the sow at farrowing, fed either standard lactation diet (Cont) or those supplemented with 21 g/day MgSO_4_ (Mg).

	Cont	Mg	Significance
Inter-piglet birth interval (min)	17.1 ± 3.0	19.0 ± 2.7	NS
Farrowing duration (h)	2.4 ± 0.4	3.6 ± 0.4	*p* < 0.05
Number of total born piglets	13.0 ± 0.6	12.5 ± 0.6	NS
Number of piglets born alive	12.3 ± 0.6	11.7 ± 0.6	NS
Stillborn piglets (%)	5.5 ± 1.9	5.9 ± 1.9	NS

s.e.m: standard error of the mean.

**Table 3:** Mean ± s.e.m inter-piglet birth interval, cumulative birth duration, and cord blood measurement of lactate, pH, and glucose for piglets classed as heavy (>1.8 kg), average (0.8 kg to 1.8 kg), or light (<0.8 kg), first (1 to 4), middle (5 to 8), or last (>9) in the birth order, and those with no meconium staining (score 0) to those heavily stained (score 3).

		Birth Interval (min)	Cumulative Duration (min)	Cord Lactate (mmol/L)	Cord pH	Cord Glucose (mmol/L)
Weight	Heavy (n = 56)	21.7 ^a^ ± 4.5	80.0 ^a^ ± 25.7	9.6 ^ab^ ± 1.4	7.5 ^a^ ± 0.2	1.7 ^a^ ± 0.4
	Average (n = 313)	7.4 ^b^ ± 3.6	60.5 ^b^ ± 24.7	10.1 ^a^ ± 1.4	7.3 ^ab^ ± 0.2	1.4 ^ab^ ± 0.4
	Light (n = 17)	11.3 ^ab^ ± 6.6	57.4 ^b^ ± 27.7	8.1 ^b^ ± 2.0	7.1 ^b^ ± 0.3	0.7 ^b^ ± 0.6
	*Significance*	*p* < 0.01	*p* < 0.001	*p* < 0.01	*p* < 0.01	*p* < 0.10
Order	First (n = 117)	-	9.0 ^a^ ± 15.5	7.0 ^ab^ ± 1.3	-	2.1 ^ab^ ± 0.4
	Middle (n = 157)		75.5 ^b^ ± 15.0	6.9 ^a^ ± 1.3		2.5 ^a^ ± 0.4
	Last (n = 125)		148.9 ^c^ ± 15.2	7.5 ^b^ ± 1.3		2.0 ^b^ ± 0.4
	*Significance*	NS	*p* < 0.001	*p* < 0.01	NS	*p* < 0.05
Stain score	0 (n = 112)	-	45.2 ^a^ ± 24.7	9.4 ^a^ ± 1.3	7.4 ^a^ ± 0.2	1.6 ^ab^ ± 0.4
	1 (n = 129)		55.6 ^ab^ ± 24.4	9.5 ^a^ ± 1.3	7.4 ^a^ ± 0.2	1.7 ^ab^ ± 0.4
	2 (n = 70)		69.9 ^ab^ ± 24.7	10.0 ^ab^ ± 1.3	7.4 ^a^ ± 0.2	1.5 ^a^ ± 0.5
	3 (n = 75)		75.7 ^b^ ± 24.8	11.0 ^b^ ± 1.3	7.1 ^b^ ± 0.2	2.1 ^b^ ± 0.4
	*Significance*	NS	*p* < 0.05	*p* < 0.05	*p* < 0.05	*p* < 0.05

^a,b^ Means in a column, within measure, not having the same superscript are significantly different (*p* < 0.05).
